# ARS-Media for Excel: A Spreadsheet Tool for Calculating Media Recipes Based on Ion-Specific Constraints

**DOI:** 10.1371/journal.pone.0166025

**Published:** 2016-11-03

**Authors:** Randall P. Niedz

**Affiliations:** United States Department of Agriculture, Agricultural Research Service, U.S. Horticultural Research Laboratory, Fort Pierce, Florida, United States of America; Beijing Forestry University, CHINA

## Abstract

*ARS-Media for Excel* is an ion solution calculator that uses “Microsoft Excel” to generate recipes of salts for complex ion mixtures specified by the user. Generating salt combinations (recipes) that result in pre-specified target ion values is a linear programming problem. Excel’s Solver add-on solves the linear programming equation to generate a recipe. Calculating a mixture of salts to generate exact solutions of complex ionic mixtures is required for at least 2 types of problems– 1) formulating relevant ecological/biological ionic solutions such as those from a specific lake, soil, cell, tissue, or organ and, 2) designing ion confounding-free experiments to determine ion-specific effects where ions are treated as statistical factors. Using *ARS-Media for Excel* to solve these two problems is illustrated by 1) exactly reconstructing a soil solution representative of a loamy agricultural soil and, 2) constructing an ion-based experiment to determine the effects of substituting Na^+^ for K^+^ on the growth of a Valencia sweet orange nonembryogenic cell line.

## Introduction

Calculating a mixture of salts (‘recipe’) to generate exact solutions of complex ionic solution mixtures is required for at least 2 types of problems–

### 1. Formulating Relevant Ecological/Biological Ionic Solutions

Reconstructing the ionic composition of ecological and biological fluid samples such as those from a specific lake, soil, cell, tissue, or organ requires a method to determine the type and concentration of salts to use to formulate the recipes. Some examples of studies that attempted to reconstruct these types of compositions include fresh water lakes [1], *in vitro* growth and production of the glycoalkaloid solamargin [2], micropropagation of yellow passion fruit on a medium based on analysis of healthy young leaves [3], soil solution-based microbial culture medium [4], conifer suspension medium based on seed composition [5], micropropagation of hazelnut on a medium based on seed composition [6], *in vitro* rooting of the carob tree on a medium based on leaf composition [7], micropropagation of *Galanthus* species on a medium based on bulb composition [8], micropropagation of Persian walnut on a medium based on *in vitro* explant composition [9], micropropagation of almond, *Prunus dulcis*, based on the mineral composition of raw almond kernels [10], shoot organogenesis of *Eucalyptus dunnii* based on the mineral composition of young stump shoots [11]. No examples were found where the resulting recipe reconstructed the composition precisely. A well-documented example of the difficulty of formulating complex ionic solutions is the study of Angle et al. [4], who reported the development of a growth medium based on the ionic composition of a soil solution representative of “loamy agricultural soils with a neutral pH”. They explain that most culture media formulations for culturing soil organisms “do not resemble the environment from which the microorganisms were isolated”, and this creates the possibility that “both genetic and phenotypic properties of microorganisms growing on these media may be different from those of the same microorganisms growing in soil.” Their extensive manipulations to recreate the soil solution are well-described, but the soil solution was only approximated, not recreated. The authors stated, “Although this medium is a much better approximation of the composition of the soil solution than previous media, concentrations of several ions deviate from those in the soil solution more than we had hoped.”

### 2. Design of Ion Confounding-Free Experiments to Determine Ion-Specific Effects

Determining the effects of specific ions such as NO_3_^-^, NH_4_^+^, PO_4_^3-^, K^+^, SO_4_^2-^, Ca^2+^, Mg^2+^, and Cl^-^ requires experiments designed where ions rather than salts are treated as statistical factors or mixture components. Because ion-based experiments are free of ion-confounding, ion-specific effects can be determined [12, 13]. Varying ions that are not part of the experiment results in ion-confounding. This occurs when the objective of the experiment is to determine the effects of a specific ion(s) but salts are varied. For example, determining the effect of K^+^ by varying KCl confounds the effect of K^+^ with the effect of Cl^-^ and the effect of the interaction of K^+^ x Cl^-^. Because the treatments in ion-based experiments are various combinations of ions, each treatment requires a recipe of the salts necessary to achieve these combinations. Recipes are required to actually setup the experiment. Constructing these recipes “by hand” is quite difficult and probably why nearly all reports on the effects of specific ions use salts, and consequently exhibit ion confounding.

For example, a recent paper by Krishnasamy et al. [14] tried to determine the effects of K^+^ and Na^+^ on four wheat cultivars that varied in their differential productivity on sodic and K-deficient soils. The objective was to improve K fertilization on these soils based on the K-use efficiency of the cultivar. The study varied the 2 salts KCl and NaCl to determine the specific effects of K^+^ and Na^+^. This approach confounded the effects of K^+^ and Na^+^ with those of Cl^-^ (Table 1). The specific effects of K^+^ with Na^+^, or their interaction with Cl^-^, cannot be determined. Further, using only Cl^-^ to determine the effects of K^+^ with Na^+^ assumes that the anion used, other than contributing a charge, has no effect. Are the effects of K^+^ with Na^+^ the same regardless of the anion used? Would the effects of K^+^ with Na^+^ be the same if NO_3_^-^, SO_4_^2-^, or acetate^-^ were varied rather than Cl^-^? If the specific effects of K^+^ with Na^+^ cannot be determined, what can be determined from this type of study? The two salts are the independent factors so the main effects of KCl and NaCl, and their interaction, KCl x NaCl, can be determined. The study compares salts, not ions. The conclusions of the study about the specific effects of K^+^ and Na^+^ may be true, but such conclusions cannot be derived from the experiment. Unfortunately, unlike this study, many studies provide insufficient or unclear information needed to deconstruct the treatments into their ion compositions [15, 16]. Designing an experiment to determine the effects of K^+^ and Na^+^ requires varying only K^+^ and Na^+^ and holding constant all other ion levels. Including various anions as factors or components would extend the experiment.

**Table 1:** Deconstructing the experiment of Krishnasamy et al. [14] to determine the effects of K^+^ and Na^+^ into the actual ion levels. The experiment varied three ions K^+^, Na^+^, and Cl^-^. The effects of K^+^ and Na^+^ are confounded with those of Cl^-^.

	Salts varied		Final Ion Composition			Total mM	
Treatments	KCl	NaCl	K^+^	Na^+^	Cl^-^	∑ mM	pH^*^
	mM	mM	mM	mM	mM	mM	
1	0.54	0	0.54	0	0.54	1.08	5.6
2	0.54	1.1	0.54	1.1	1.64	3.28	5.6
3	0.54	2.2	0.54	2.2	2.74	5.48	5.6
4	0.54	4.3	0.54	4.3	4.84	9.68	5.6
5	0.54	8.7	0.54	8.7	9.24	18.48	5.6
6	1.34	0	1.34	0	1.34	2.68	5.6
7	1.34	1.1	1.34	1.1	2.44	4.88	5.6
8	1.34	2.2	1.34	2.2	3.54	7.08	5.6
9	1.34	4.3	1.34	4.3	5.64	11.28	5.6
10	1.34	8.7	1.34	8.7	10.04	20.08	6.9

^*^ Calculated using the chemical equilibrium software MINEQL^+^ [17].

This paper describes the utility, validation, and availability of an ion solution calculator, *ARS-Media for Excel* that uses “Microsoft Excel” to generate recipes of salts that, when dissolved, result in the complex ion mixtures specified by the user.

## Materials and Methods

*ARS-Media for Excel* uses “Microsoft Excel (2013)” (herein referred to as Excel) to solve Eq (1) to generate recipes of salts for complex ion mixtures specified by the user.

$$minimize\;c_{1}X_{1}+c_{2}X_{2}+\ldots +c_{n}X_{n}$$ (1)

where *c* is a known weighting coefficient, and *X* is the unknown amount of an individual salt,

$$\begin{array}{l} subject\;to\;a_{i1}X_{1}+a_{i2}X_{2}+\cdots +a_{in}X_{n}(\leq ,=,\geq )\;b_{i}(i=1,2,\ldots \;m)\\ \;X_{1},\ldots \;X_{n}\geq 0 \end{array}$$

where *a* are known coefficients of ion proportionality for each salt *X* that contributes an ion defined by the target ion concentration *b*; a non-negative constraint ensures positive values. Eq (1) results in a list of the type and amount of salts to achieve the exact ion concentrations specified by *b*.

*ARS-Media for Excel* provides the universality and convenience of a spreadsheet but with additional functionality compared to an earlier Window-based, *ARS-Media*, desktop application [12]. The added functionality is a result of “Microsoft Excel’s” Solver Add-in program (Frontline Systems, USA) and its more extensive set of options. For example, forcing Eq (1) to include specific salts at specified concentrations in the final recipe is now possible. The calculations of using Eq (1), with examples, is described in detail by Niedz and Evens (2006) [12]. The spreadsheet is formula-based and uses no macros because they sometimes conflict with institutional IT security systems. *ARS-Media for Excel* is free and available by download, along with instructions, from the U.S. Department of Agriculture, Agricultural Research Service software site as ARS-Media for Excel.

### Utility and Validation

The utility of *ARS-Media for Excel* is that it solves 2 problems common in experimental biology, 1) formulating relevant ecological/biological ionic solutions, and 2) design of ion confounding-free experiments to determine ion-specific effects. An example of using *ARS-Media for Excel* to solve each of these problems is provided.

#### Formulating relevant ecological/biological ionic solutions

An exact formulation of the soil solution that Angle et al. [4] attempted to construct, as described above, was calculated using *ARS-Media for Excel*. The ion values entered into *ARS-Media for Excel* are those listed in Table 1 of the Angle et al. [4] paper (Table 2).

**Table 2:** Ionic compositions of the “soil solution equivalent” (SSE) and *ARS-Media for Excel* formulation and how each compares to the ion levels of the target soil solution.

Ion	Soil solution	SSE	% Deviation of SSE from soil solution	*ARS-Media for Excel* formulation	% Deviation of *ARS-Media for Excel* from soil solution
	mM	mM		mM	
NO_3_	4.7	2.5	- 47	4.7	0
NH_4_	0.4	2.5	+ 525	0.4	0
HPO_4_	0.005	0.005	0	0.005	0
Na	1.4	2.5	+ 79	1.4	0
Ca	12.4	4.0	- 68	12.4	0
Mg	3.4	2.0	- 41	3.4	0
K	0.3	0.503	+ 68	0.3	0
Cl	1.9	4.0	+ 111	1.9	0
SO_4_	3.4	5.0	+ 47	3.4	0
Fe	0.02	0.2	+ 900	0.02	0

#### Design of ion confounding-free experiments to determine ion-specific effects

To illustrate the use of *ARS-Media for Excel* for ion experimentation, an experiment free of ion confounding to determine the specific effects of K^+^ and Na^+^ on the growth of a Valencia sweet orange nonembryogenic cell line was conducted.

### Tissue Source

**Fig 1:**
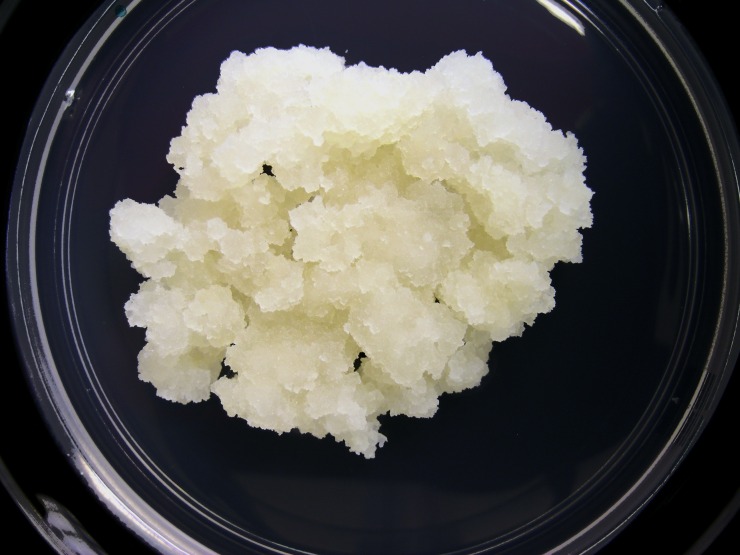
Nonembryogenic citrus cell line of Valencia sweet orange.

A nonembryogenic cell line of Valencia sweet orange was developed and maintained as described [18] (Fig 1). Briefly, the line was subcultured every 30 days onto MT medium [19] supplemented with 1-μM 2,4-dichlorophenoxyacetic acid (2,4-D), 1-μM 6-benzylaminopurine (BA), and 100 mg l^−1^ casein hydrolysate. MT medium includes the inorganic salts of MS medium [20]. The cultures were grown in a growth cabinet under low light (15–20 μmol m^−2^ s^−1^), provided by cool-white fluorescent lamps, constant 27°C, and a 4-h photoperiod.

### Experimental Design

The experiment was a 2-component quadratic mixture design with D-optimal point selection (Table 2). Mixture designs vary the proportion of the components but fix the amount. Thus, the approach was to determine the effect of substituting K^+^ with Na^+^ on fresh weight growth of a nonembryogenic cell line of Valencia sweet orange. The cell line was grown on MS salts where the proportion of [K^+^] and [Na^+^] were varied while 1) maintaining the MS [K^+^ + Na^+^] of 23.2 mM, and 2) holding all other ions of MS medium constant. The resulting experiment varied only K^+^ + Na^+^ and therefore free of ion confounding (Table 3). A total of 23.2 mM was selected because the total [K^+^ + Na^+^] of MS medium is 20.25 mM. However, adjusting pH under our conditions to 5.8 using NaOH requires an additional 2.95 mM Na^+^. Thus, adding 2.95 mM Na^+^ results in formulations with the target pH of 5.8 without the pH adjustment step. The final design included 13 points (3 model, 2 lack-of-fit, and 8 replicates to estimate pure error) and was sufficient to model a quadratic polynomial.

**Table 3:** The five unique formulations that comprised the experiment. The experiment was free of ion confounding because only the target ions Na+ and K+ were varied. All other ions in MS medium were held constant.

Media	Na^+^	K^+^	NH_4_^+^	NO_3_^-^	PO_4_^3-^	Ca^2+^	Mg^2+^	SO_4_^2-^	Cl^-^	∑ mM
	mM	mM	mM	mM	mM	mM	mM	mM	mM	mM
1	*23*.*2*	*0*	20	40	1.25	3	1.5	1.6	6	96.55
2	*17*.*4*	*5*.*8*	20	40	1.25	3	1.5	1.6	6	96.55
3	*11*.*6*	*11*.*6*	20	40	1.25	3	1.5	1.6	6	96.55
4	*5*.*8*	*17*.*4*	20	40	1.25	3	1.5	1.6	6	96.55
5	*0*	*23*.*2*	20	40	1.25	3	1.5	1.6	6	96.55

One response was measured, fresh weight growth. The initial amount of tissue cultured onto each dish was approximately 0.68 g (± 0.07 S.D.). The tissue was cultured for 14 days, the final weight measured, and the fresh weight growth calculated as the percent increase over the initial culture weight. Six culture dishes were used to estimate the response, as an average, at each treatment design point (Table 1). Thus, the experiment utilized a total of 78 culture dishes (13 treatment design points x 6 dishes per design point). A polynomial model and ANOVA were generated. Model adequacy tests were conducted as described by Anderson and Whitcomb (2005) [21] and included an analysis of the normal probability plot of studentized residuals to determine if data transformation was required. Data transformation was not required. The statistical software used was Design Expert® 9 (Stat-Ease, Inc, Minneapolis, MN).

*ARS-Media for Excel* calculated the recipes for the 5 unique formulations (3 model and 2 lack-of-fit points) that comprised the experiment. The mM ion levels for each of the 5 formulations used in the design (Table 3) were entered into *ARS-Media for Excel* and recipes calculated using Excel’s Solver function (Table 4). To simplify the illustration, not all of the inorganic mineral nutrient ions in MS medium were included in the calculations. The ions Cl^-^, Fe^3+^, Mn^2+^, Zn^2+^, BO_3_^3-^, I^-^, Cu^2+^, MoO_4_^2-^, and Co^2+^ were added to each formulation using the salts and amounts used for MS medium.

**Table 4:** Recipes generated by *ARS-Media for Excel* for the 5 media listed in Table 2. In addition to the salts listed, all recipes included the MS salts that deliver Fe^3+^, Mn^2+^, Zn^2+^, BO_3_^3-^, I^-^, Cu^2+^, MoO_4_^2-^, and Co^2+^ as follows: FeSO_4_^.^7H_2_O, Na_2_EDTA^.^2H_2_O, MnSO_4_^.^4H_2_O, ZnSO_4_^.^4H_2_O, H_3_BO_3_, KI, CuSO_4_^.^5H_2_O, Na_2_MoO_4_^.^2H_2_O, and CoCl_2_^.^6H_2_O. These salts did not vary between formulations.

		Media				
		1	2	3	4	5
Salts	K^+^ (mM)	23	17.3	11.6	5.9	0.2
	Na^+^ (mM)	0.2	5.9	11.6	17.3	23
		mg/L	mg/L	mg/L	mg/L	mg/L
(NH_4_)_2_SO_4_		13	-	-	-	-
CaCl_2_.2H_2_O		441	441	441	441	441
KH_2_PO_4_		170	51	51	51	27
KNO_3_		2199	1711	1135	559	-
MgSO_4_.7H_2_O		370	370	370	370	370
Na_2_SO_4_		-	14	14	14	14
Na_3_PO_4_		-	143	143	143	143
NaH_2_PO_4_		-	-	-	-	21
NaNO_3_		-	261	746	1230	1700
NaOH		8	-	-	-	-
NH_4_NO_3_		1461	1601	1601	1601	1601
NH_4_OH		54	-	-	-	-

## Results

### Formulating Relevant Ecological/Biological Ionic Solutions

Using *ARS-Media for Excel* to solve for the combination of salts to construct a recipe for the specified soil solution (Table 5) resulted in a formulation (Table 6) that exactly achieves each of the soil solution ion levels (Table 5). The *ARS-Media for Excel* solution is exact.

**Table 5:** Treatment design points and fresh weight growth data of a 2-component quadratic mixture design. The proportions of K^+^ and Na^+^ were varied while the total amount was kept constant at 23.2 mM, the level in MS medium. For example, treatment point #1 would include 17.4 mM Na^+^ and 5.8 mM K^+^.

Treatment Design Points	Na^+^	K^+^	Fresh weight growth
	proportions		%
1	0.75	0.25	1038
2	0.5	0.5	1082
3	1	0	217
4	1	0	196
5	0.5	0.5	1317
6	0.25	0.75	1031
7	0.75	0.25	906
8	0	1	867
9	1	0	215
10	0	1	1125
11	0.5	0.5	1021
12	0	1	1146
13	0.25	0.75	1209

**Table 6:** Recipe generated by *ARS-Media for Excel* to recreate the soil solution ion composition of Angle et al. [4].

Salts	Amount
	mg L^-1^
(NH_4_)_2_SO_4_	26.43005
Ca(OH)_2_	16.67094
CaSO_4_*2H_2_O	391.7074
K_2_SO_4_	26.139
Mg(NO_3_)_2_*6H_2_O	602.6105
MgCl_2_*6H_2_O	193.164
MgSO_4_*7H_2_O	24.64998
Na_2_FeEDTA*2H_2_O	8.521155
Na_2_HPO_4_	0.7098
Na_2_SO_4_	95.8902

### Design of Ion Confounding-Free Experiments to Determine Ion-Specific Effects

Tissue was harvested, weighed, and the difference between the final and initial fresh weights calculated to determine the percent increase in fresh weight. The ANOVA revealed significant linear and quadratic effects (Table 7).

**Table 7:** ANOVA of the effect of K^+^ and Na^+^ on fresh weight growth of citrus nonembryogenic cells. The coefficients for K^+^ and Na^+^ under the linear mixture are estimates of the response at each vertex, not estimates of the effects of these two ions. The K^+^ x Na^+^ term is not an interaction term, though it looks like one, but a quadratic blending term unique to mixture models. This term is used to determine if the mixture components exhibit nonlinear blending and if that blending is synergistic or antagonistic.

Source	Sum of Squares	df	Mean Squares	F Value	p-value Prob > F	Coefficient Estimate
Model	1.70E+06	2	8.51E+05	45	<0.0001	
Linear mixture	1.01E+06	1	1.01E+06	54	<0.0001	
K^+^						1011
Na^+^						251
K^+^ x Na^+^	6.92E+05	1	6.92E+05	37	0.0001	2112
Residual	1.87E+05	10	1.87E+04			
Lack of Fit	6.53E+04	2	3.26E+04	2.14	0.1804	
Pure Error	1.22E+05	8	1.53E+04			
Cor Total	1.89E+06	12				

**Fig 2:**
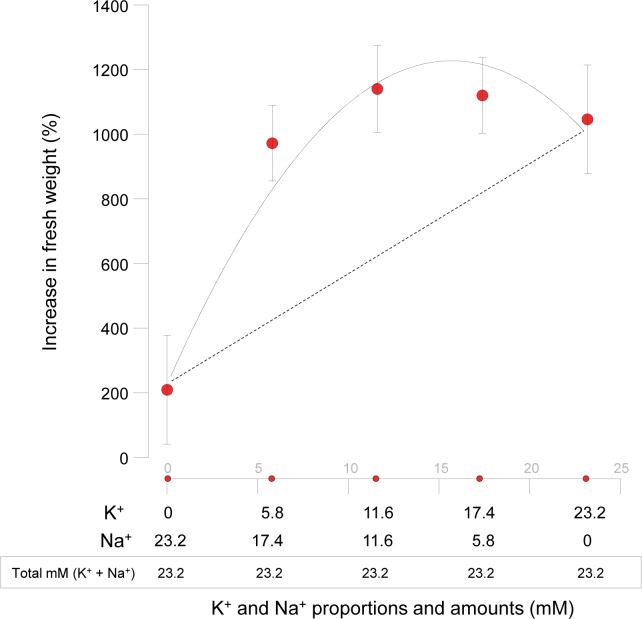
Response plot of the effect of the 2-component K^+^ and Na^+^ mixture on % increase in fresh weight of Valencia sweet orange nonembryogenic callus. The significant linear component, depicted by the dotted line, is the estimated response at each vertex (251% and 1011%), and the significant quadratic component indicates nonlinear blending or that the response deviates (above) from what would be predicted by the linear component. However, the graphic reveals a sharp reduction in fresh weight at 0 mM K^+^ / 23.2 mM Na^+^ and essentially a flat relationship between 5.9 mM K^+^ / 17.3 mM Na^+^ to 23 mM K^+^ / 0.2 Na^+^. This indicates a threshold where Na^+^ cannot completely substitute for K^+^.

The plotted response surface shows a sharp reduction in fresh weight at 0 mM K^+^ / 23.2 mM Na^+^ (Fig 2), revealing that for *in vitro* growth of this tissue Na^+^ cannot completely substitute for K^+^, a result consistent with the view that Na^+^ can only substitute for K^+^ for certain nonspecific functions [22]. From 5.9 mM K^+^ / 17.3 mM Na^+^ to 23 mM K^+^ / 0.2 Na^+^ the response is essentially flat, indicating 1) Na^+^ can partially substitute for K^+^ and 2) a threshold where Na^+^ cannot completely substitute for K^+^.

## Discussion

*ARS-Media for Excel* addresses two common problems in experimental biology. The first is how to exactly formulate ionic solutions of ecological/biological relevance. These types of solutions are relevant to virtually every aspect of biological and agricultural science, from plant mineral nutrition to ecological compositions in nature to the ionic makeup of spinal fluid. The study by Angle et al. [4] and their precise and extensive description of their attempt to construct a recipe to replicate the ionic composition of a soil solution representative of a loamy agricultural soil with a neutral pH illustrates the difficulty of solving, or attempting to solve, this type of problem by hand. *ARS-Media for Excel* solves this problem.

The second problem is how to design an experiment that can determine ion-specific effects. *ARS-Media for Excel* was used to determine the ion-specific effects of K^+^ and Na^+^ on the growth of a sweet orange nonembryogenic cell line. The conclusions were similar to what others have concluded about the relationship between K^+^ and Na^+^. Wakeel et al. (2015) [22] in their review of the substitution of potassium (K^+^) by sodium (Na^+^) explain that K^+^ is “the only monovalent cation which is essential for all higher plants”. However, for certain nonspecific metabolic functions, such as osmoregulation, Na^+^ can substitute for K^+^. Understanding the substitution of K^+^ with Na^+^ is of practical importance as a potential tool to address the increasing problem of soil salinity, crop production, and the differential nutritional needs of plants and animals for Na^+^ [22]. However, experiments to determine the effects of K^+^ with Na^+^ on plant growth are generally confounded. To the best of my knowledge, this seemingly simple experiment represents the only example of an experiment free of ion-confounding to quantify the effects of K^+^ and Na^+^ that are free of the confounding influence of co-varied ions. Designing experiments free of ion confounding should either confirm and/or clarify current knowledge of an ion’s effects, or reveal new and possibly unexpected effects, particularly those related to interactions, synergies, and antagonisms.

The two problems are not mutually exclusive but are closely related. After formulating an ecological/biological ionic solution the next logical step is to determine the ions in the solution that have the largest effects on an organism’s growth. One example is to first formulate a water body where toxic algae blooms regularly occur followed by ion-based designs determine the extent to which mineral nutrients affect the growth of toxic algae species. A second example is developing *in vitro* growth media based on the ionic composition of a “healthy” cell/tissue/organ/plant. Ion-based experiments would use the ionic composition to construct and orient the design space.

Calculating salt recipes without using the linear programming algorithm described by Eq (1) is extremely difficult and, for practical purposes, not possible, and salt recipes generated “by hand” are only rough approximations of the target ion levels [4]. *ARS-Media for Excel* makes these calculations trivial, thus making these types of experiments possible. Designing experiments based on ions is almost nonexistent in the literature but is necessary to understanding what the ions effects, and more importantly, their interactions with other ions. This type of information would complement well the current advances in molecular biology and omics.
